# Nephrology in Veterinary Medicine

**DOI:** 10.34067/KID.0000000000000273

**Published:** 2023-10-16

**Authors:** Jonathan Daniel Foster

**Affiliations:** Nephrology and Urology, Friendship Hospital for Animals, Washington, DC

**Keywords:** AKI, chronic kidney failure, dialysis, glomerular disease, hemoperfusion, leptospirosis, nephrotoxicity, peritoneal dialysis, renal transplantation, transplantation

## Abstract

Veterinary nephrology is a specialized field of veterinary medicine providing a high level of care for animals with all types of kidney disease. Veterinarians complete extensive training to become board-certified in veterinary nephrology–urology. Companion animal nephrology is the most advanced field; however, all species are afflicted by a variety of renal disorders. Most naturally occurring animal kidney diseases have similar disorders found in people; where veterinary research is lacking, clinical management is often modified from standard of care in people. Veterinarians have become adept at scaling down procedures to safely perform them on dogs and cats weighing only a few kilograms. Advanced diagnostics (renal biopsy, cystoscopy, fluoroscopic studies, *etc.*) and therapeutics (renal replacement therapy, interventional endourology, *etc.*) are commonly performed within the practice of veterinary nephrology–urology. Collaboration between veterinary and human nephrologists may advance both disciplines and improve care for people and animals alike.

## Introduction

Modern veterinary medicine is a diverse profession that shares many similarities to human medicine. Most veterinarians are general practitioners—primary care physicians who provide routine wellness and preventative care and often the initial assessment for both acute and chronic diseases.^1^ Most (84.8%) veterinarians enter clinical practice, and the majority provide care to companion animals (dogs and cats). Only 4% of clinical veterinarians have a mixed animal practice (in the style of James Herriot's *All Creatures Great and Small*) where they also treat horses and production animals. Fifteen percent of veterinarians work in academia or corporate domain where they do not primarily perform patient care.

The daily activities of a veterinarian in clinical practice are quite variable. Most general practitioners will see appointment consultations and perform minor surgical procedures. Emergency veterinarians typically do not have any scheduled appointments because they need flexibility to quickly triage and manage patients presenting through the emergency service. Veterinary specialists also perform scheduled consults and elective and emergency procedures as dictated by their field of expertise.

## Education, Training, and Ongoing Maintenance/Update of Expertise

Entrance to veterinary school is competitive because there are only 33 accredited programs in the United States. Throughout veterinary school, students are taught basic medicine in many species and then may shift their focus to the species they intend to treat after graduation. To become a specialist in a particular field of veterinary medicine, additional training is needed. Acceptance into residency training is also quite competitive because the number of applicants greatly exceeds the number of available training positions in nearly all fields.

### Veterinary Medical Training

There is not a global standard in veterinary education. Instead, each country determines their own, which may be influenced by available resources, public opinion on animal welfare, expectations of pet owners, and more. This can create significantly different approaches to veterinary care between countries. In the United States, veterinary medical education often tends to follow a similar track to that of human medical school. Veterinary medical school education begins after students graduate undergraduate college (although some veterinary programs may enroll exceptional students after completion of 3 years of undergraduate school). Veterinary school takes 4 years to complete. Some countries, such as Australia and the United Kingdom, have a combined program where students complete undergraduate and veterinary courses and graduate with a veterinary degree in 5 or 6 years.

Veterinary school education bears much similarity to human medical school education with the major difference being training in the major anatomic and physiologic differences between multiple commonly treated veterinary species (companion animals, equids, food and fiber species). Elective courses are typically offered to provide study of zoo or wildlife species for those students with particular interest. Clinical problem solving, surgical principles, and exposure to specialty medicine occurs in the earlier portion of veterinary school with entry to clinical rotations typically occurring during the senior year (although some schedule variations exist between schools). During their clinical year, students examine patients, develop diagnostic and treatment plans, and communicate with clients although graduate veterinarians (often boarded specialists) are the primary care providers. Many veterinary schools allow their students select an academic major that will allow them to focus on specific species (small animal, large animal, food animal, equine, wildlife/exotics, *etc.*) in their junior and senior year of school.

On graduation of veterinary school and passing a national accreditation examination, veterinarians are licensed to practice all aspects of veterinary medicine. While some procedures (spinal surgery, endoscopy, *etc.*) may be unethical to perform by those who have not received adequate training, there is no legal hindrance to them doing so.

### Postdoctoral Medical Training

Optional advanced medical training can be pursued by graduate veterinarians, including general rotating internships, specialized internships, and specialty residency and fellowship training. Internship training is an optional year of mentored training that graduate veterinarians may pursue, similar to postgraduate year 1 for human physicians. Throughout this year, interns are primary care providers to hospitalized animals, receive patients through emergency department, and work alongside boarded specialists to assist them in their care of patients within that specific service. Companion animal internships occur in academic teaching and private specialty hospitals. In 2023, 171 hospitals offered such internship training programs—ranging from 1 to 16 interns per program.

Some veterinarians enter internship training as part of their path to pursue specialty training and board certification. However, many veterinarians will enter primary care practice after the internship with an increased level of expertise and comfort in their ability to diagnose and treat disease in that setting.

There are 22 officially recognized specialty colleges, offering board certification in 46 specialty fields.^2^ The American College of Veterinary Nephrology and Urology (ACVNU) is the newest specialty college, receiving provisional approval in March 2022. Specialty residency training is typically 3 years in duration, although some 2-year programs exist. In this time, residents learn detailed pathophysiology and gain experience practicing within their specific specialty field. After completion of residency requirements (often including performing and publication of original research) and passing one or more board examinations, veterinarians are now recognized as a diplomat of their respective college and a specialist within the field.

### Nephrology Training

The specialized field of veterinary nephrology began in the 1950s; Dr. Don Lowe is considered the grandfather of veterinary nephrology and began training veterinary graduate students in the discipline. Drs. Carl Osborne and Ken Bovée were early experts in this developing field and created additional training programs that produced numerous nephrology-focused internists. Basics of nephrology and urology are taught within small animal internal medicine and critical care residency training. However, those veterinarians who want to gain expertise in challenging nephrology cases, extracorporeal therapies, and endourology would have to seek additional training through externships, training workshops, and self-study. Recognizing that internal medicine and critical care residency training did not provide complete or thorough training in nephrology, experts in the field began work to develop nephrology–urology as an established veterinary specialty. In 2022, the American Board of Veterinary Specialists approved the creation of the ACVNU.

The ACVNU began training its first resident class in 2023 and expects the first diplomats to receive board certification in 2025. Residency training involves mastery of both upper and lower urinary tract disorders. Common nephrology–urology conditions treated in veterinary medicine are listed in Table 1. The ACVNU has created several pathways for veterinarians to obtain advanced training. Traditional nephrology–urology residency training can occur with both in-house and remote mentorship training. In these early stages of the ACVNU, there are not enough training programs to accommodate all resident candidates. The remote training option allows residency program directors to mentor residents at multiple hospitals, thus increasing training availability. Residents who perform direct patient care (internists, criticalists, cariologists, *etc.*) are eligible for full diplomat status; those who perform nonpatient care (radiologists, pathologists, *etc.*) are eligible for associate diplomat status. Residency training involves a core curriculum of 150 hours of lecture, provided by topic experts from around the world. These lectures are delivered live and allow an opportunity for all residents and lecturers to interact. Lectures are recorded to facilitate dyssynchronous viewing. Publication of primary research is a prerequisite to earn board certification.

**Table 1:** Common veterinary nephrology–urology disorders

• AKI
• Amyloidosis
• Bacterial cystitis
• Chronic tubulointerstitial disease
• Focal segmental glomerulosclerosis
• Immune complex GN
• Juvenile and congenital renal disorders
• Micturition disorders
• Mineral and bone disorders of CKD
• Nephrotic syndrome
• Nephroureteral and urethral obstruction
• Prostatitis
• Pyelonephritis
• Systemic hypertension
• Tubular disorders
• Urinary incontinence
• Urolithiasis
• Urothelial carcinoma

Kidney biopsy acquisition and interpretation is taught and is a commonly used tool in the evaluation of proteinuric dogs and cats. Unless the patient has very small kidneys, tissue is obtained through percutaneous ultrasound-guided needle biopsy. Most veterinary nephrologists submit kidney biopsies directly to the International Veterinary Renal Pathology Service; nephropathologists interpret light, immunofluorescent, and electron microscopy on these submitted samples to provide a structural diagnosis.^3–7^

Extracorporeal therapies have increased availability throughout the world; in part attributable to a 13-month training course offered by the Hemodialysis Academy. The Hemodialysis Academy is taught by an international group of veterinary nephrologists who have expertise in extracorporeal blood purification. Lectures and discussion sessions are held through videoconference with recordings of lectures made available for trainees to view at their own time. Over the past decade, several 100 veterinarians have received certification through this course; many have started programs offering extracorporeal blood purification (an updated list of veterinary units can be found at www.queenofthenephron.com). The ACVNU has developed guidelines for fellowship training in extracorporeal therapies. Candidates would train in intermittent hemodialysis (IHD), continuous and prolonged renal replacement, apheresis, and cytapheresis procedures along with their various applications in veterinary medicine.

The second fellowship currently recognized by the ACVNU is endourology. Candidates would study cystoscopic and minimally invasive endourologic procedures to manage urolithiasis, urogenital neoplasia, nephroureteral and urethral obstruction, urinary incontinence, and other micturition disorders.

After completion of residency training, individuals may prefer to focus on nephrology or urology, although many will see similar frequency of both areas. The more renowned veterinary nephrology–urology hospitals have very strong programs in both disciplines.

### Maintenance and Updating of Expertise

Several special interest groups exist within veterinary nephrology—the American Society of Veterinary Nephrology and Urology and its European counterpart are the most popular. Here any veterinarian with an interest in these fields can participate in case discussions and online rounds. The International Renal Interest Society is a veterinary organization chartered with advancing education on kidney disease throughout the world. They have created staging/grading systems for both AKI and CKD as well as produced numerous educational articles freely available on their website (www.iris-kidney.com).

Providing 2 ½ days of lecture and a day of hands-on laboratories, the International Renal Interest Society Renal Week conference is the preeminent continuing education event within veterinary nephrology. Experts and trainees from around the world gather at this biannual conference. Colleagues from human nephrology are frequently invited to speak and collaborate. Similarly, veterinary nephrologists regularly attend and have presented research abstracts at the American Society for Nephrology's Kidney Week conference.

The American Society of Veterinary Nephrology and Urology organizes online videoconference-based journal clubs and renal pathology rounds. ACVNU residents attend a monthly journal club to discuss seminal manuscripts as well as recent landmark studies in both human and veterinary nephrology. Some of the more influential peer-reviewed journals in the field include the *Journal of Veterinary Internal Medicine*, *Journal of Veterinary Emergency and Critical Care*, *Journal of the American Veterinary Medical Association*, *American Journal of Veterinary Research*, and *Journal of Small Animal Practice*.

## The Practice of Veterinary Nephrology

Veterinary nephrologist–urologists perform a wide variety of diagnostic and therapeutic procedures. Most work in multispecialty hospitals—in both academia and private practices. A typical workday involves providing care for hospitalized patients, seeing appointment consults, and performing procedures. Most procedures are elective; however, patients with AKI or urinary obstruction may require emergent intervention.

### Consultations and Common Diagnoses

Most nephrology consults are scheduled for an hour to provide the veterinarian sufficient to review the patient's medical history, perform an examination, obtain more historical and symptomatic information from the pet owner, and to request necessary diagnostics. Some nephrologists separate their consult and procedure days, while others perform both every day. Because most veterinary nephrologists work at large 24-hour referral hospitals, they also have primary case management of hospitalized patients. Common diseases that are seen through outpatient consult service include CKD, tubular disorders (such as Fanconi Syndrome), glomerular disease (many lack histopathologic diagnosis and are classified as protein-losing nephropathy), polyuric syndromes, and electrolyte disorders. They also will manage patients with AKI—both as inpatients and as consults during their recovery. Veterinary nephrologists also manage (and some prefer) urologic diseases including urinary incontinence, urolithiasis, and urinary tract infections.

### Specialty Procedures

#### Extracorporeal Therapies—Renal Replacement and Therapeutic Apheresis

**Figure 1:**
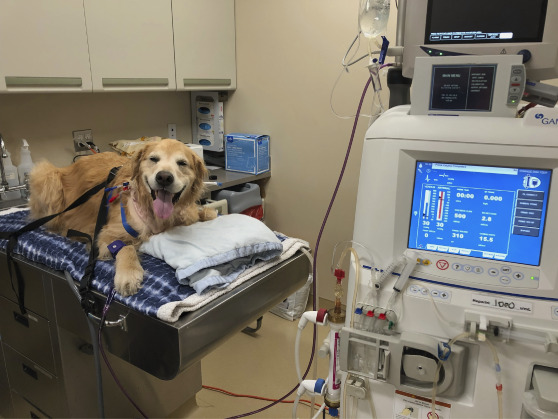
**An 8-year-old golden retriever about to receive IHD for the management of advanced CKD.** The patient is awake and will be fed throughout the treatment. They wear a harness fastened to the table with seatbelts to prevent them from pulling out their catheter. IHD, intermittent hemodialysis.

**Figure 2:**
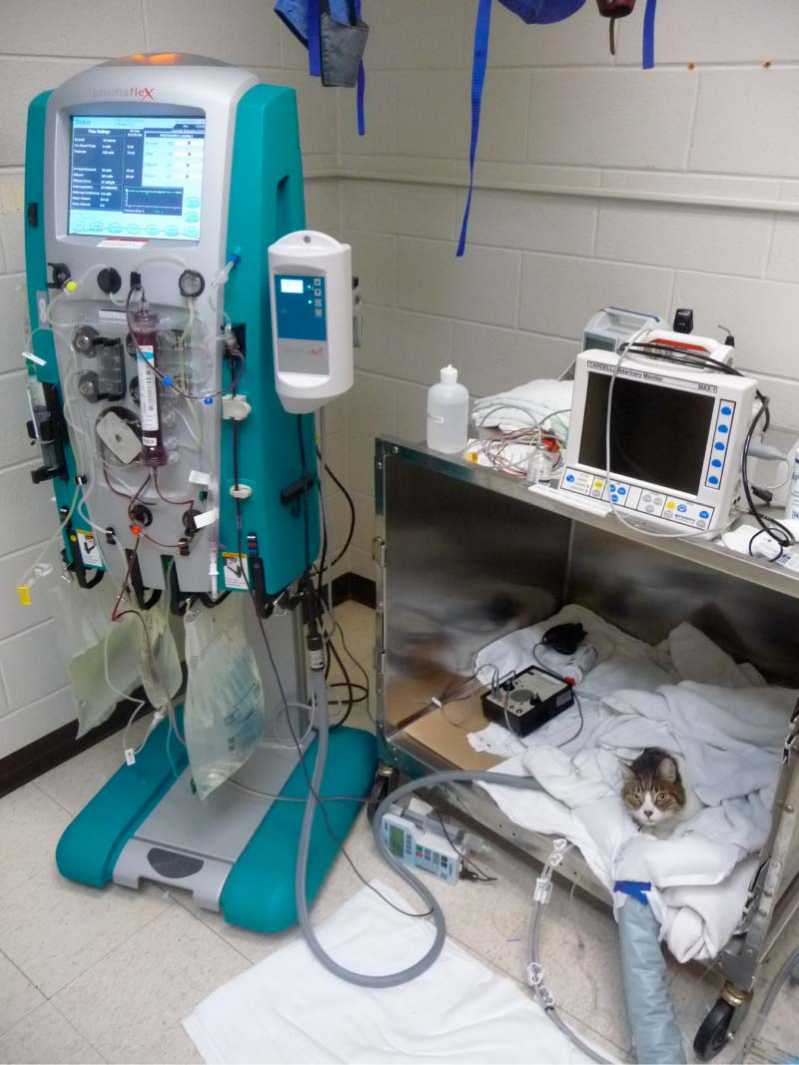
**A 7-year-old domestic shorthair cat receiving continuous renal replacement therapy for the management of AKI.** Patients generally receive constant monitoring throughout the treatment by veterinary nurses and the attending nephrologist.

**Figure 3:**
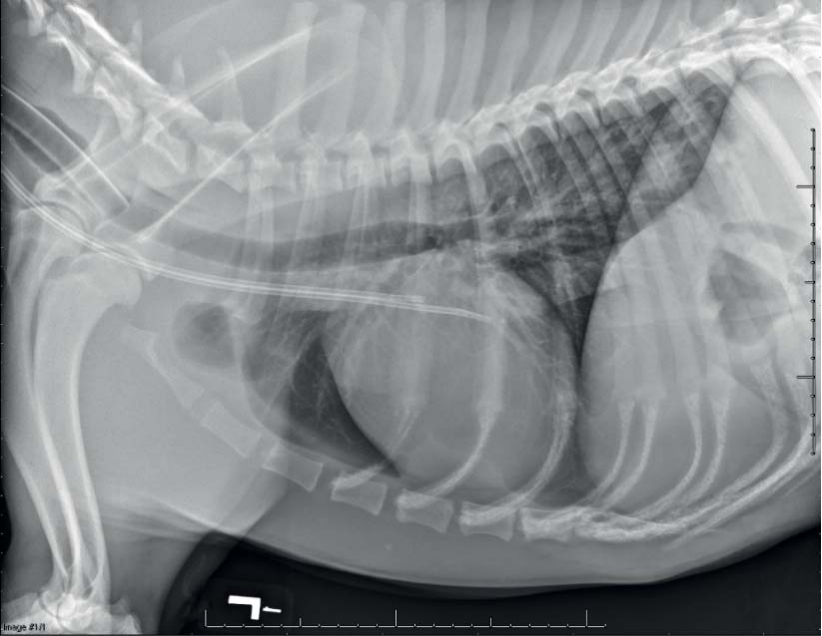
**A lateral thoracic radiograph demonstrating optimal placement of a split-tip dual lumen hemodialysis catheter.** The catheter has been placed in the right external jugular vein, and the tip of the catheter is within the right atrium. The patient has been anesthetized for catheter placement; and endotracheal tube is also visible.

Extracorporeal therapies have been performed in companion animals for more than 50 years. In the first few decades, availability was very limited and typically used IHD (Figure 1).^8–12^ Continuous renal replacement and prolonged intermittent renal replacement have now become commonplace alongside IHD (Figure 2).^13–16^ Treatments are performed using a dedicated dual lumen catheter in the external jugular vein (Figure 3).^17^ Most animals treated with extracorporeal renal replacement (ERRT) are being managed for severe AKI. The goal is to improve their quality of life and allow time for their kidneys to heal and regain function. Some patients may receive daily ERRT and then be transitioned to twice- or thrice-weekly outpatient IHD to manage their disease. Success of renal recovery is dependent on the etiology of AKI, and most animals will have some degree of CKD after an episode of AKI.^18^ The client cost of ERRT is quite substantial; each ERRT treatment is >$1000 at most veterinary hospitals in the United States. Pet insurance is available; however, the coverage is not standardized between providers. The author has performed 67 IHD treatments on a cat who developed AKI associated with chemotherapy for multiple myeloma; insurance reimbursed the client 90% of costs for all treatments. Dogs with CKD can be managed with chronic outpatient dialysis, but client costs can be quite substantial. Dogs have been treated for over a year with outpatient IHD, providing a quality of life that could not be otherwise obtained. Pet owners are generally satisfied with the improvement seen in their pet's symptoms when ERRT is initiated. Other client-owned animal species have been treated with ERRT, including goats, sheep, cows, and a tortoise. Horses have been treated with both peritoneal dialysis and ERRT.^19–21^ Owing to their large size, average clearance rates in horses is quite low (average kt/V of 0.31); to increase clearance, two dialysis machines have been used each with two dialyzers operating in parallel. Zoo animals and wildlife are rarely treated with ERRT because of challenges of obtaining vascular access, risks of sedation, and need for repeated treatment.

Owing to their comfort and expertise with renal replacement therapies, nephrologists are typically the providers delivering apheresis. Therapeutic plasma exchange has been a commonly used adjunctive therapy for many immune-mediated disorders in dogs, including immune-mediated hemolytic anemia and thrombocytopenia, myasthenia gravis, polyradiculoneuritis, and immune-complex GN.^22–26^

**Figure 4:**
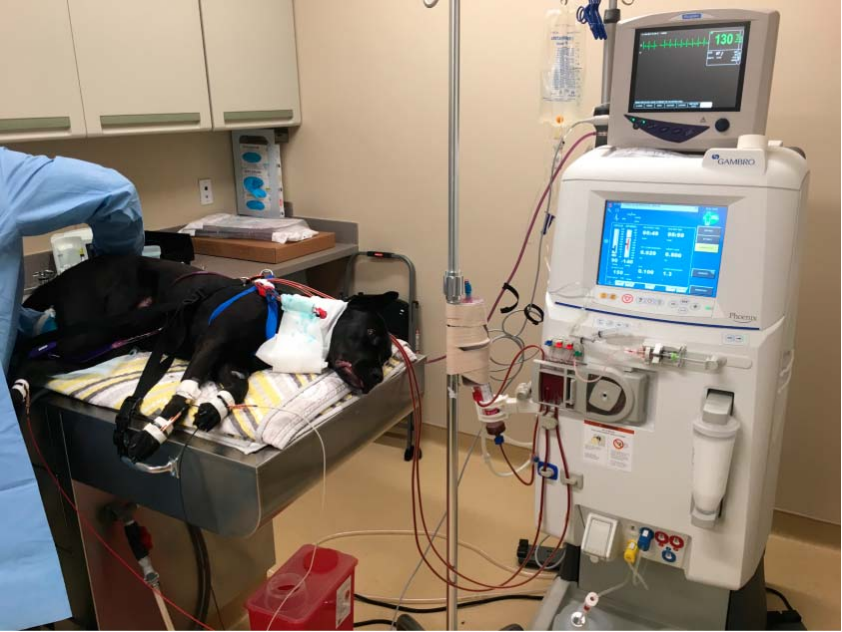
**A 3-year-old Labrador retriever who ingested >1200 mg/kg of ibuprofen.** The patient is comatose at the start of charcoal hemoperfusion. Approximately 40 minutes into treatment, the patient was awoke and was able to walk back to their cage at the completion of a 3-hour treatment. Despite the massive intoxication, acute kidney and liver injuries were avoided with the use of ECTR in this patient. ECTR, extracorporeal toxin removal.

Extracorporeal toxin removal (ECTR) has become central to the management of acute intoxications in dogs and cats.^27–29^ Nonsteroidal anti-inflammatory drugs (NSAIDs) are the most commonly treated intoxication. Ibuprofen and naproxen have serious adverse effects in dogs and cats and are not used in veterinary medicine.^30–36^ Dogs may often eat all tablets within a bottle (sometimes the bottle as well) resulting in a significant overdose. NSAID toxicity commonly results in AKI, acute liver injury, gastrointestinal ulceration, neurologic depression, coma, and death. ECTR can prevent significant organ dysfunction and death when initiated soon enough (Figure 4). It is now recognized as a safe and effective therapy along with traditional management. Charcoal hemoperfusion often used to treat toxicities with an appropriate pharmacokinetic profile.^37–44^ The first veterinary-specific extracorporeal platform is a hemoperfusion machine (Aimalojic); it may be used by many emergency hospitals that do not have an extracorporeal therapies service, thus increasing the availability of ECTR throughout the United States.

While some nephrotoxins, such as NSAIDs and ethylene glycol may be removed by ECTR and patients may avoid substantial AKI, other toxins are not amenable to blood purification. Lily plants are exquisitely nephrotoxic to cats—all portions of the plant are toxic.^45–50^ Cats will often develop severe AKI with anuria lasting 3–14 days. Even with extracorporeal renal support, it remains challenging to prevent overhydration and hyperkalemia; prognosis remains guarded. Grapes, raisins, and currants are associated with AKI in dogs; however, the mechanism is unknown.^51–57^ Recently, the tartaric acid has been suggested to be the toxic component but remains to be confirmed.^58,59^ The sensitivity may be dependent on the type of grape or perhaps susceptibility within the dog. The author has dialyzed dogs with anuric AKI after ingesting as few as ten grapes.

Leptospirosis is frequently encountered in dogs, although the prevalence is regional throughout the United States.^60,61^ Clinical disease typically involves severe AKI, cholestatic acute liver injury, and thrombocytopenia.^62,63^ Some patients also develop pulmonary hemorrhage.^64–66^ Despite severe disease, survival for dogs with leptospirosis is generally favorable.

#### Kidney Transplantation

Kidney transplantation is an available option for cats and may be less expensive than performing chronic dialysis in a cat.^67–71^ After transplantation, cats are maintained with prednisolone and cyclosporine throughout the rest of their life. Historically, it has been very challenging to provide adequate immunosuppression to dogs to provide tolerance toward the graft without resulting in an increased risk of opportunistic infections.^67,72,73^ Kidney transplantation is performed by veterinary surgeons after extensive training. Only three universities offer feline kidney transplantation in the United States: University of Pennsylvania, University of Wisconsin, and University of Georgia. Most programs require the pet owner to adopt the donor cat who provided the kidney to their pet.

#### Peritoneal Dialysis

Peritoneal dialysis is performed less frequently, despite its low tech requirements.^74–78^ Some veterinarians may opt to perform peritoneal dialysis in patients with small body weight because of the risk of hemodynamic instability associated with a large extracorporeal blood volume with ERRT. Cats have an average weight of 5 kg and blood volume of 300 ml. Peritoneal dialysis has been used in cats to help manage their AKI.^77,79^ However, complications are very common including hypoalbuminemia, dialysate retention, subcutaneous leakage of dialysate, abdominal pain, and septic peritonitis. Alternatively, use of a packed red blood cell prime of the extracorporeal circuit can be used to safely treat small patients with ERRT despite the large extracorporeal circuit volume.^80^

There are no veterinary-specific machines that are used to perform ERRT; veterinary nephrologists in the United States typically are using the Phoenix, AK98, Prismax, and Prismaflex machines from Baxter as well as the Fresenius 2008 T. Patients as small as 1.2 kg have been treated with ERRT. The extracorporeal circuit volume often exceeds 20% of the patient's blood volume, necessitating the use of circuit priming with colloid or packed red blood cells. Hypotension, hypothermia, and hypoxemia are among the more common complications encountered during ERRT in animals. Patients are monitored closely throughout treatment, typically with a trained veterinary technician observing a maximum of three patients simultaneously. Central venous oxygen saturation monitoring is commonly performed on veterinary patients to help prevent such complications. Dialysis disequilibrium syndrome has been observed in cats and dogs; symptoms include disorientation, vocalization, seizures, and acute death.^9,81^ To help prevent such consequences, initial ERRT treatments will deliver conservative solute clearances, and prophylactic administration of mannitol is often given during the first few treatments. Several veterinary hospitals have obtained a Medtronic CARPEDIEM machine to perform ERRT in small patients. Some have modified the circuit of the CARPEDIEM to perform plasma exchange safely in patients as small as 1.5 kg. Plasma exchange is performed on larger dogs with the Spectra Optia, Fresenius Amicus, or Prismax/Prismaflex systems.

#### Interventional Endourology

In companion animals, minimally invasive endourology has replaced open surgical procedures whenever possible. Interventional radiology is a developing field within veterinary medicine, and many nephrologist–urologists perform minimally invasive endourologic procedures.

Cystoscopy is routinely performed in dogs, cats, and large animals.^82^ Veterinary-specific cystoscopes are not available; however, flexible ureteroscopes used for humans are commonly used for male dog cystoscopy (urethral diameter ranges between 2.5 and 18 French). Ureteroscopy is possible in dogs after passive ureteral dilation with ureteral stent placement.^83^ Ureteral and urethral stenting is commonly performed for benign and malignant obstructions.^84–88^ Endoscopic sclerotherapy is performed for patients with primary (idiopathic) renal hematuria.^89,90^ Both intracorporeal and extracorporeal shock wave lithotripsy is used to manage urolithiasis.^91–93^

## Clinical Vignette

**Figure 5:**
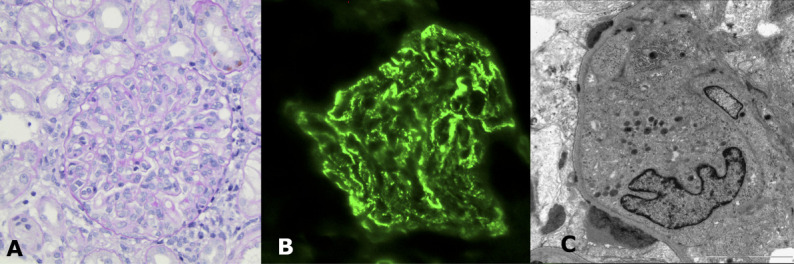
**Renal histopathologic findings of immune complex proliferative GN in a 3-year-old neutered male cockapoo.** (A) ×40 magnification, PAS stain: The glomerulus is severely hypercellular, and glomerular capillary loops are distended by the presence of cells. Endocapillary hypercellularity is due to the presence of circulating mononuclear cells and neutrophils. Mesangial hypercellularity is also present. (B) C3 immunostaining: diffuse, global moderate granular labeling of capillary walls. TEM: subepithelial immune complexes are present within this glomerulus (others have subendothelial and mesangial deposits). Notable, some deposits resemble humps seen in postinfectious proliferative GN in people. Infectious disease screening in this patient was negative. PAS, periodic acid–Schiff; TEM, transmission electron microscopy.

A 3-year-old castrated male cockapoo was evaluated for acute lethargy and anorexia. Initial lab work identified azotemia (BUN 164 mg/dl [reference range (RR), 7–27 mg/dl], creatinine 4.5 mg/dl [RR 0.5–1.8 mg/dl]), hyperphosphatemia (17.4 mg/dl [RR, 2.5–6.8 mg/dl]), and hypoalbuminemia (2.4 g/dl [RR, 2.6–3.9 g/dl]). He was proteinuric with a spot urine protein:creatinine ratio of 8.6 (RR <0.2) and also hypertensive (systolic BP, 192 mm Hg). While he was initially referred for renal replacement therapy, a primary glomerular disorder was suspected. An ultrasound-guided percutaneous needle biopsy was performed and submitted for complete evaluation. The patient was estimated to have a low risk of complications with ERRT (body weight 15.7 kg, estimated blood volume 1.25 L), but there was no emergent need for correction of electrolyte or acid-base abnormalities. Instead, immunosuppressive therapy (prednisone 2 mg/kg by mouth every 24h and mycophenolate mofetil 5 mg/kg by mouth every 12h) was initiated in the interim. The results of renal histopathology were diagnostic of immune-complex proliferative GN (Figure 5). The results of infectious disease testing (leptospirosis, bartonellosis, borreliosis, *etc.*) were negative, and immunosuppressive therapy was continued for presumptive primary immune complex proliferative GN. Telmisartan was also administered at 1.3 mg/kg by mouth every 24h. Recheck lab work performed 1 month later and found resolution of the azotemia (serum creatinine 1.5 mg/dl, BUN 22 mg/dl) and hyperphosphatemia (4.3 mg/dl). His proteinuria was significantly improved (urine protein:creatinine ratio [UPC], 0.88). The prednisone was tapered and discontinued over 4 weeks while mycophenolate was continued for 5 months. Repeat kidney biopsy was recommended but declined. The proteinuria was mild and stable throughout this time, and the patient remained nonazotemic. Four months after the discontinuation of mycophenolate, a presumptive relapse of GN occurred with the patient again developing azotemia (BUN 169 mg/dl, creatinine 6.6 mg/dl) and worsening of proteinuria (UPC, 12.8). The patient was reinduced with prednisone and mycophenolate mofetil with a similar positive response becoming nonazotemic (BUN 32 mg/dl, creatinine 1.3 mg/dl) and minimally proteinuric (UPC 0.48) within a month. Mycophenolate was discontinued after an additional 6 months of treatment, and telmisartan was continued. The patient has not demonstrated further relapse and continues to be nonazotemic and minimally proteinuric up to the time of manuscript submission (6 years later).

## One Health Nephrology

Veterinary nephrologists have long collaborated with their human counterparts. This mutually beneficial relationship began as a natural shared interest in medicine but now can be viewed in the larger scope of One Health Nephrology. Veterinarians often struggle to find funding to perform research, and human nephrology too often relies on rodent models of disease, which may not be accurate representations of disease in people. By studying naturally occurring disease in veterinary species, both human and veterinary nephrologists gain insight into pathophysiology and treatment of disease. Such arrangements create new clinical trials in animals—opportunities for patients to have new control of their disease, as well as generate preclinical data that can guide trials in people. Pet owners are often eager to participate in clinical trials. The hope of new treatments and the desire to give back to the pet community are strong motivators for clients to enroll their pets in studies. Human nephrology far exceeds its veterinary counterpart regarding the volume of new research produced yearly. Although veterinarians have long attended American Society of Nephrology Kidney week, fewer nephrologists have attended or participated in veterinary conferences. Such collaborations produce mutual benefits to both parties and should be encouraged whenever possible. Formal establishment of One Health Nephrology workgroups may help secure funding and increase research productivity.

Some therapeutics widely used for people (sodium-glucose cotransporter-2 inhibitors, rituximab and other monoclonal antibodies, immunomodulatory drugs, *etc.*) are prohibitively expensive, untested, or ineffective in veterinary patients, leaving a void of some treatment options. Clinical trials in veterinary patients as part of preclinical drug investigation provide the opportunity for veterinarians to gain insight in new therapeutics and allows for discovery of drug effects in nonrodent species.

## Future of Veterinary Nephrology

Veterinary nephrology has advanced significantly over the past 30 years with development of more specific diagnostics and refinement of therapeutic procedures. This momentum reached an inflection point with the establishment of the ACVNU and onset of formal nephrology–urology training. As the college grows, we expect advancements in the assessment and management of disease as well as an increase in scholarly research. It is an exciting time to be part of the veterinary nephrology community. We welcome colleagues who treat bipedal mammalian animals to strike up conversations, collaborations, and working relationships to benefit all: physician, veterinarian, pet owner, and pet.
